# The complete mitochondrial genome of *Labeo catla* (Hamilton, 1822) using long read sequencing

**DOI:** 10.1080/23802359.2020.1870879

**Published:** 2021-02-09

**Authors:** Bismay Sahoo, Gargee Das, Lakshman Sahoo, Kanta Das Mahapatra, Prem K. Meher, Uday Kumar Udit, Jitendra Kumar Sundaray, Paramananda Das

**Affiliations:** Fish Genetics and Biotechnology Division, ICAR-Central Institute of Freshwater Aquaculture, Bhubaneswar, India

**Keywords:** *Labeo catla*, mitgenome, Oxford nanopore sequencing, Cyprinidae

## Abstract

*Labeo catla* is a widely cultured species in monoculture and polyculture systems of the Indian subcontinent. In this study, the complete mitochondrial genome sequence of catla was reconstructed from Oxford Nanopore sequence data. The mitochondrial genome is 16,600 bp in length (accession no. is MN830943) which is larger than the previously reported catla mitogenomes. Like other vertebrate mitochondrial genomes, it has 13 protein-coding genes, 22 tRNAs, 2 rRNAs and a putative control region. Most of the mitogenes are encoded on H-strand. Phylogenetic analysis showed that *Labeo catla* is more closely related to *Labeo rohita* than other labeo species. The catla mtgenome reported here will facilitate population genetics, phylogenetics and molecular taxonomy of Indian major carps.

*Labeo catla* (Hamilton, 1822) commonly known as ‘catla’ is one of the Indian major carp and the second most popular freshwater aquaculture species in the Indian subcontinent due to its fast growing nature and appealing taste. Mitochondrial DNA has been widely used to trace the geographic distribution of genetic variation due to its maternal inheritance and a high mutation rate (Avise et al. 1987). In comparison with partial mtDNA sequences, complete mtDNA sequence has numerous advantages, and it can provide novel frame of reference on teleost phylogenies (Miya et al. 2003). The complete mitochondrial genome of catla has been sequenced and reported using Sanger’s and Illumina sequencing (Accession no.– NC016892, JQ087872 and KY419138). Although the advantages of the NGS technology were explored by the previous study through Illumina sequencing technology (Sahoo et al. 2017), here we have reconstructed the complete mitochondrial genome of catla (Accession no. – MN830943) from Oxford Nanopore reads for the first time and compared with complete mitogenomes of other cyprinids.

Total genomic DNA was extracted from testis tissue of catla stored at ICAR-CIFA (Voucher no. CIFA-CC01), India (20.1861° N, 85.8564° E) using standard phenol chloroform method (Sambrook and Russel 2001). Nanopore libraries were prepared from genomic DNA from one individual and sequenced on Oxford Nanopore (MinION) platform. A total of 20 GB sequence data was obtained. *De novo* assembly was performed with default parameters using MaSuRCA-3.2.8. Contigs ranging from 10 to 16 kb in length were subjected to blast in NCBI database. The longest contigs of 16,600 bp in size was found to be the mitogenome of catla and was annotated using MitoAnnotator (Iwasaki et al. 2013). The complete mitogenome sequences for other cyprinids were downloaded from the NCBI database. A maximum likelihood phylogenetic tree was constructed by taking 13 complete cypriniformes mitogenome, one siluriformes mitogenome as outgroup along with the reconstructed catla mitogenome using MEGA X (Kumar et al. 2018).

In this study, the complete mitogenome of catla is 16,600 bp in length with GeneBank accession no. MN830943 and composed of 13 protein coding genes, 22 tRNAs, 2 rRNAs and a putative control region. The mitogenome of catla reconstructed from Nanopore data is largest in size in comparison to the mtDNAs reported earlier, which shows 2 and 1 bp variation in D-loop and intergenic spacer region in between tRNA^Asn^ and tRNA^Cys^, respectively. Organization of genes in the catla mitogenome is similar to other vertebrates. Except for few genes, the most of the mitogenes are encoded in the H-strand. The overall base composition of catla mitogenome is as follows: A: 30.48% G: 14.63% C: 28.09% T: 26.78% and A + T content is 57.26%. Mostly, in all the protein coding genes, ATG is used as start codon except CO1 gene, which encodes GTG. Likewise, TAA and an incomplete stop codon T_–_ are encoded by protein-coding genes except for ND6, which uses TAG in catla. Out of 22 tRNA genes, 21 tRNA genes of catla can fold into a typical cloverleaf structure, except for tRNA^Ser^(AGY) lacking dihydrouridine arm identified by the online software tRNAscan SE 2.0 (Chan and Lowe 2019). The size of tRNA genes varied from 67 to 76 bp. The D-loop is 931 bp in size and contains a microsatellite (TA)_7_, a putative termination-associated sequence and three conserved sequence blocks. There are 6 overlaps and 14 intergenic spacer regions with a 37 bp long intergenic spacer region in between tRNA^Asn^ and tRNA^Cys^. Fascinatingly, the mitochondrial genome yielded from the present study showing 99% resemblance to the mitochondrial genome sequenced earlier in our laboratory (Bej et al. 2012; Sahoo et al. 2017) having 16,594 and 16,597 bp length, respectively. The phylogenetic analysis inferred that *Labeo catla* (Figure 1) is closely related to *Labeo rohita*. The complete mitogenome sequence of *L. catla* will facilitate population genetics and evolutionary studies of cyprinidae family.

**Figure 1.**
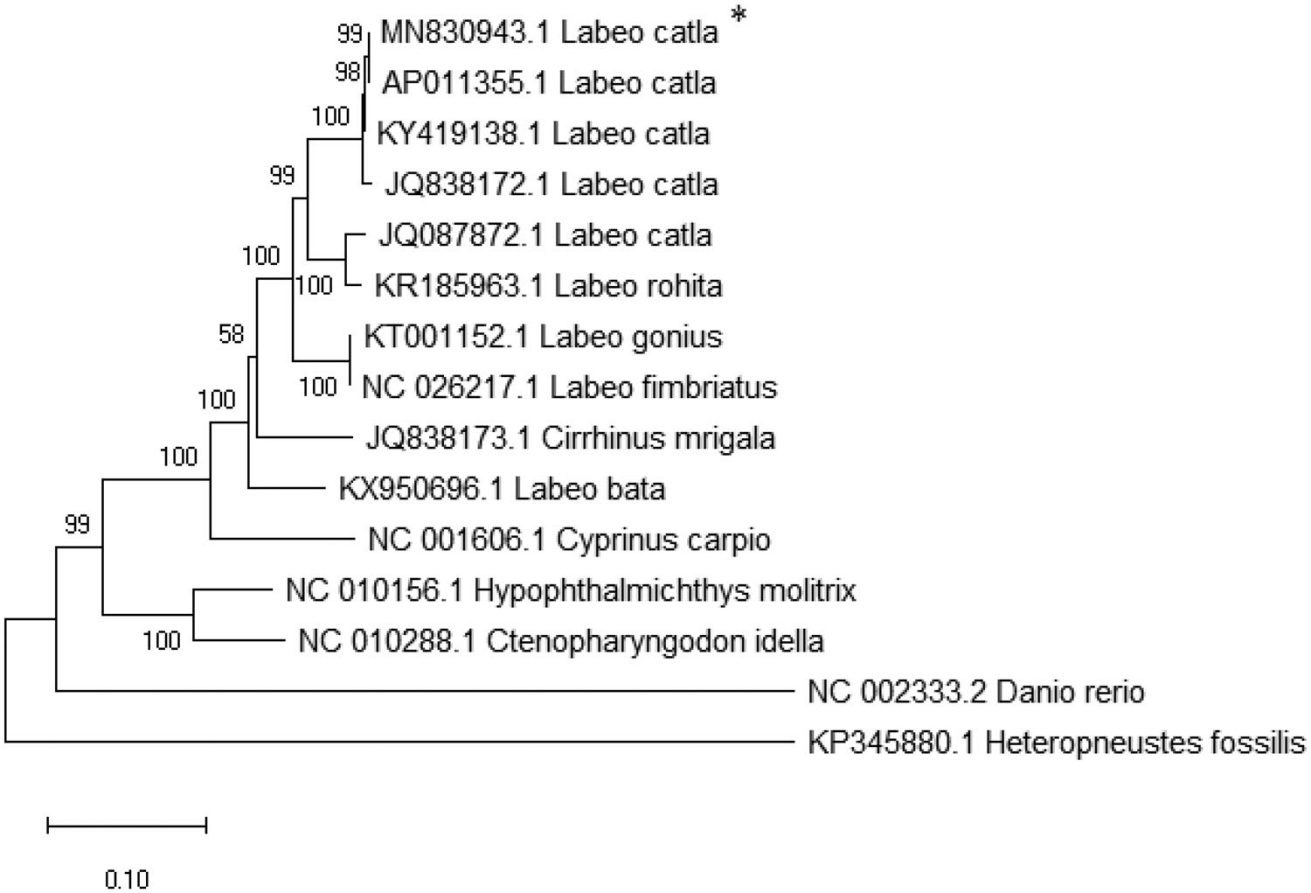
ML tree of complete mitogenome sequences of 15 teleosts (*present study).

## Data Availability

The data that support the findings of this study are openly available in “NCBI” at https://www.ncbi.nlm.nih.gov/, reference number MN830943.

## References

[CIT0001] Avise JC, Arnold J, Ball RM, Bermingham E, Lamb T, Neigel JE, Reeb CA, Saunders NC. 1987. Intraspecific phylogeography: the mitochondrial DNA bridge between population genetics and systematics. Annu Rev Ecol Syst. 18(1):489–522.

[CIT0002] Bej D, Sahoo L, Das SP, Swain S, Jayasankar P, Das PC, Routray P, Swain SK, Jena JK, Das P. 2012. Complete mitochondrial genome sequence of *Catla catla* and its phylogenetic consideration. Mol Biol Rep. 39(12):10347–10354. 23086264 10.1007/s11033-012-1912-5

[CIT0003] Chan PP, Lowe TM. 2019. tRNAscan-SE: searching for tRNA genes in genomic sequences. Methods Mol Biol. 1962:1–14. 31020551 10.1007/978-1-4939-9173-0_1 PMC6768409

[CIT0004] Iwasaki W, Fukunaga T, Isagozawa R, Yamada K, Maeda Y, Satoh TP, Sado T, Mabuchi K, Takeshima H, Miya M, et al. 2013. MitoFish and MitoAnnotator: a mitochondrial genome database of fish with an accurate and automatic annotation pipeline. Mol Biol Evol. 30(11):2531–2540. 23955518 10.1093/molbev/mst141 PMC3808866

[CIT0005] Kumar S, Stecher G, Li M, Knyaz C, Tamura K. 2018. MEGA X: molecular evolutionary genetics analysis across computing platforms. Mol Biol Evol. 35(6):1547–1549. 29722887 10.1093/molbev/msy096 PMC5967553

[CIT0006] Miya M, Takeshima H, Endo H, Ishiguro NB, Inoue JG, Mukai T, Satoh TP, Yamaguchi M, Kawaguchi A, Mabuchi K, et al. 2003. Major patterns of higher teleostean phylogenies: a new perspective based on 100 complete mitochondrial DNA sequences. Mol Phylogenet Evol. 26(1):121–138. 12470944 10.1016/s1055-7903(02)00332-9

[CIT0007] Sahoo L, Bit A, Meher PK, Murmu K, Sundaray JK, Das P. 2017. Rapid recovery of complete mitogenome of Indian major carp, *Catla catla* from low depth paired end Illumina sequencing. Mitochondr DNA Part B. 2(1):155–156. 10.1080/23802359.2017.1298413 PMC7799541 33473750

[CIT0008] Sambrook J, Russel DW. 2001. Molecular cloning: a laboratory manual. Cold Spring Harbor (NY): CSH Laboratory Press.

